# Control of light absorbance using plasmonic grating based perfect absorber at visible and near-infrared wavelengths

**DOI:** 10.1038/s41598-017-02847-1

**Published:** 2017-06-01

**Authors:** Duc Minh Nguyen, Dasol Lee, Junsuk Rho

**Affiliations:** 1 Department of Mechanical Engineering, Pohang University of Science and Technology (POSTECH), Pohang, 37673 Republic of Korea; 2 Department of Chemical Engineering, Pohang University of Science and Technology (POSTECH), Pohang, 37673 Republic of Korea

## Abstract

Conventional metamaterial absorbers have multilayer designs, where the dielectric interlayer is sandwiched between a top patterned metallic structure and bottom metallic film. Here, we demonstrate that a highly polarization-sensitive perfect absorber canbe realized by replacing the bottom metallic film with a plasmonic grating. Designs for broadband and narrowband of wavelength are proposed and numerically investigated. The designed absorbers perform high light absorption, which is above 90% over the wavelength range of 0.4–1.4 *µ*m for the broadband absorber and 98% for the absorption peak in case of the narrowband design, with a specific polarization of incident light. We find that the absorption is tunable by changing the polarization. Such absorbers offer new approach for active control of light absorbance with strong impacts for solar energy harvesting, light emitting and sensing.

## Introduction

Perfect absorber, which is a device that neither reflects nor transmits the incident light, has drawn great interest due to its importance in both science and practical applications. With the advent of metamaterials whose optical properties such as effective permittivity and permeability can be manipulated, perfect absorbers are now reported to cover all the range from microwave to visiblelight^1–3^ and can be engineered to work in both broadband and narrowband of wavelength by utilizing the extraordinary optical absorption of plasmonic resonances. While the broadband absorbers offer many promising applications in photovoltaic industry^4–6^, radar technology^7, 8^, solar cells^9^ and thermal imaging^10^, the narrowband absorbers are generally applied for sensing^11–13^ due to the selective light absorption.

Generally, metamaterial absorbers base on coupled resonators on optical cavity forming by multilayers of metal and dielectric with certain patterned structures. Many polarization-insensitive absorbers at visible and near-infrared regime have been demonstrated such as structures using mesoporous gold surfaces^14^, structures consisting of a metal-insulator-metal stack with trapezoid arrays of nanostructures patterned on the top silver film^15^ or structures based on one ultrathin layer of refractory metal^16^. Since the control of light absorbance plays a fundamental role in photonics technology with strong impact for light emitting and sensing components, metamaterial designs that can enable active control of light absorbance are necessary. Perfect absorbers that are sensitive to the polarization or incident angle therefore attract much attention, and have been realized recently by introducing anisotropy in the design structure^16–21^. For example, designs of narrowband polarization sensitive perfect absorber with botte-line and cub-like structures are proposed and demonstrated numerically^16^, and the one consisting of arrays of three-dimensional standing U-shaped resonators fabricated by two-photon polymerization followed by blanket coating of the metal is reported^21^.

In this paper, we propose a new concept of polarization sensitive perfect absorber based on plasmonic gratings that can be applied for both broadband and narrowband regimes. Comprehensive numerical study is performed for different polarizations and incident angles, and we demonstrate that high light absorption, which is above 90% over the wavelength range of 0.4–1.4 *µ*m for the broadband absorber and 98% for the absorption peak in case of the narrowband design, is achieved with only a specific polarization of incident light. By changing the polarization or incident angle, the absorption can be actively controlled and reaches minimum when the polarization is perpendicular to the one at the perfect absorbing state. This feature makes the designed absorbers very useful in controlling light absorbance, manipulating and detecting light with specific polarization.

## Theoretical background for multilayers perfect absorbers

**Figure 1:**
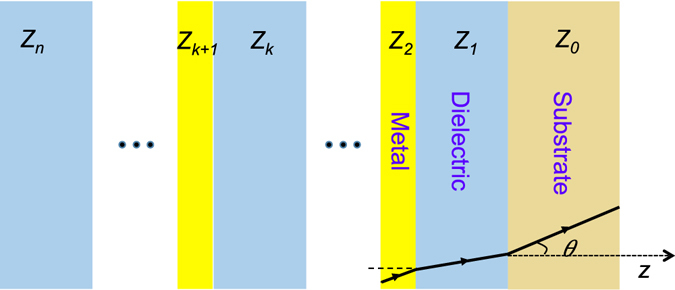
Multilayer structure for perfect absorbers. *Z*_*k*_ is the characteristic impedance of the layer medium.

We consider sandwiched multilayers of metal and dielectric as shown in Fig. 1 with layer thickness of *d*_*m*_, *d*_*d*_. In order to analyze the absorption in periodic structures, it is convenient to use the impedance transformation method ^22^ by introducing the wave impedance *Z*(*z*) = −*E*_*y*_/*H*_*x*_ of a TE wave, and the characteristic impedance of layer medium

1 $${Z}_{k}=\frac{1}{\cos \,\theta }\sqrt{\frac{{\mu }_{k}}{{\varepsilon }_{k}}},$$

where *µ*_*k*_, *ε*_*k*_ are the permeability and permittivity of the layer, respectively and *θ* is the incident angle. To deal with periodic problems, we define *Z*(*k*) as the wave impedance at the interface between layer (*k* + 1)^th^ and layer *k* ^th^. The wave impedance at the first metal/dielectric interface is

2 $$Z(1)={Z}_{1}\frac{Z(0)-i{Z}_{1}\,\tan \,{\phi }_{1}}{{Z}_{1}-iZ(0)\,\tan \,{\phi }_{1}},$$

where *ϕ*_1_ = *k*_*z*_ *d* is the phase shift of the layer, and *Z*(0) can be expressed as

3 $$Z(0)=\frac{1}{\cos \,\theta }\sqrt{\frac{{\mu }_{{\rm{substrate}}}}{{\varepsilon }_{{\rm{substrate}}}}},$$

The wave impedance for the *k* ^th^ layer can be obtained by the recurrence formula

4 $$Z(k)={Z}_{k}\frac{Z(k-1)-i{Z}_{k}\,\tan \,{\phi }_{k}}{{Z}_{k}-iZ(k-1)\,\tan \,{\phi }_{k}}.$$

The reflection of the system is finally given by

5 $$r=\frac{{Z}_{{\rm{in}}}-{Z}_{n}}{{Z}_{{\rm{in}}}+{Z}_{n}},$$

where *Z*_*in*_ is the characteristic impedance of the input medium, which is air in our case. According to the above equations, a perfect absorber can be realized by satisfying the following requirements: (i) substrate layer must reflect all the in coming light to negligee the transmission and (ii) choosing material variables such that the reflection in (5) vanishes, or in other words

6 $${Z}_{{\rm{in}}}={Z}_{n}.$$

**Figure 2:**
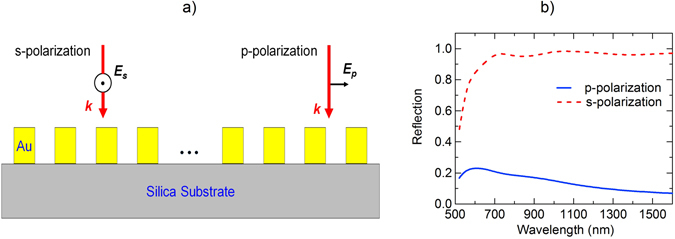
Plasmonics grating. (**a**) Gold grating on silica substrate. (**b**) Corresponding reflection for p-and s-polarized light under normal incidence.

In case the metal layer is ultra thin compared to the optical wavelength, and assume that real part of the substrate refractive index is negligible, *Z*_*n*_ in (5) will change periodically as a function of tan (*k*_*z*_ *d*_*d*_), giving rise to different absorption bands. The top *n* ^th^ layer can have a patterned metallic structure for selective light absorption based on plasmonic resonance. In conventional metamaterial absorbers, the first condition is realized by using a metallic layer as a substrate to diminish the transmission. In our work, we use plasmonic grating structure, defined as a metallic grating with period much smaller than the wavelength, at the substrate layer for reflecting selected polarization. Figure 2a presents schematic of a gold grating on silica substrate and the corresponding reflection for a grating period of 50 nm is shown in Fig. 2b. The grating has extremely high reflection (>98%) over a broad range of wavelength from visible to near-infrared (NIR) (600–1600 nm) for s-polarization incident light, but low reflection (<20%) for the p-polarization light over the same range of wavelength. We utilize this feature of the plasmonic grating for designing polarization sensitive perfect absorbers as explained in the following section.

## Broadband polarization sensitive absorber

**Figure 3:**
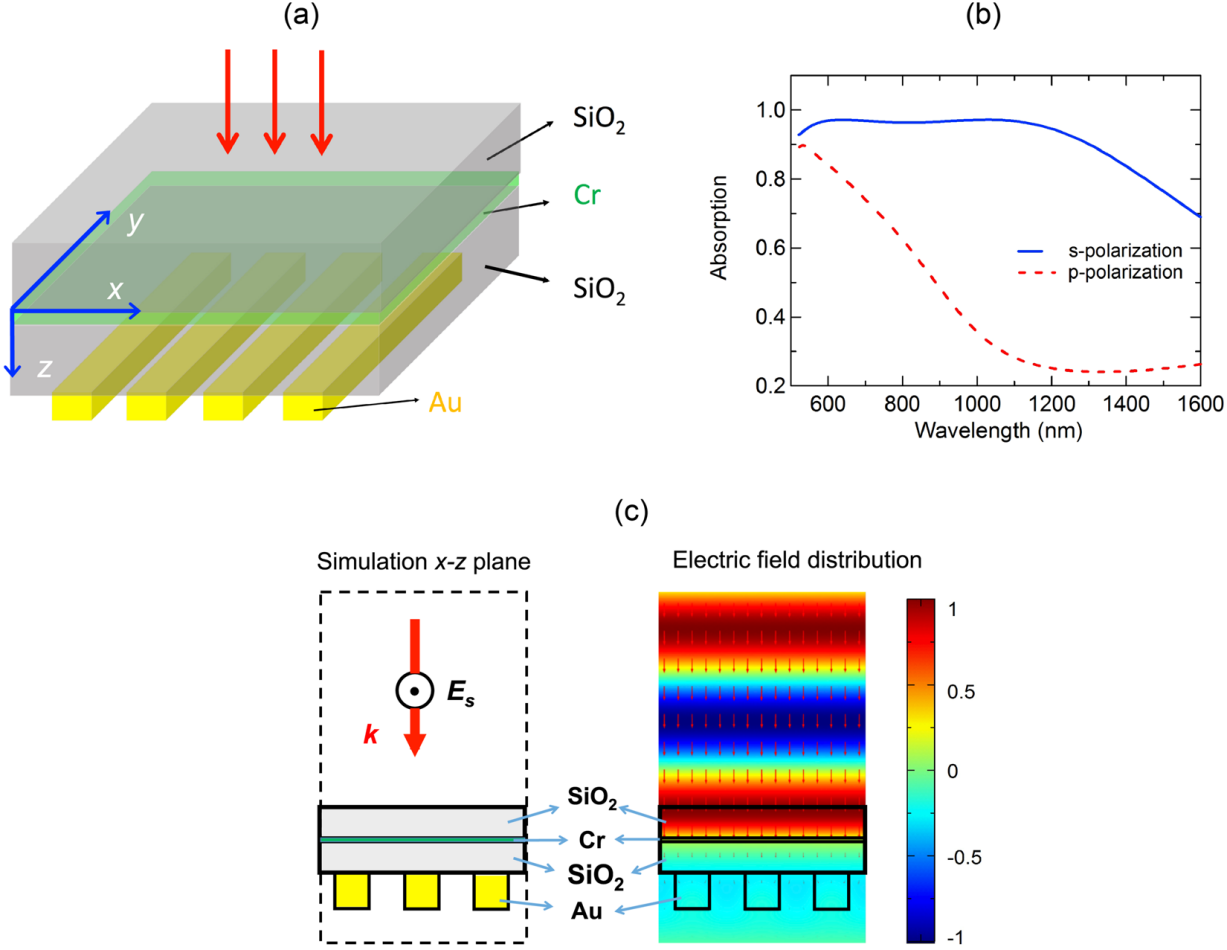
(**a**) Schematic of the broadband polarization sensitive perfect absorber. (**b**) Absorption for s-and p-polarization under normal incidence. (**c**) Corresponding field distribution at x–z plane for s-polarization at wavelength of 600 nm. The grating periodis 200 nm with filling factor of 0.5. The time-averaged power flow $\overrightarrow{P}$ (arrows in the right figure of Fig. 3c) keeps the same intensity in the air and the top silica layer but decays rapidly below the Cr layer.

Our design for broadband polarization sensitive absorber is presented in Fig. 3a. It consists of an ultra thin chromium (Cr) interlayer of 8 nm sandwiched by two silica layers of 85 nm. Gold grating structure with period of 200 nm, filling factor of 0.5 and thickness of 100 nm is deposited on one silica layer. Since the permittivity of Cr^16^ matches the ideal permittivity to satisfy the matching impedance condition (6) for broadband waveleng thin the visible and NIR, and the grating structure will reflect the light back to the silica layer for only selected polarization, the proposed structure is expected to work as a broadband polarization sensitive perfect absorber.

To demonstrate the performance of the absorber, we perform numerical investigation based on the Finite Element Method (FEM) with COMSOL and Finite Difference Time Domain method (FDTD) with Lumerical. Absorption of the proposed structure under s-and p-polarization and normal incidence are shown in Fig. 3b. When the incident light is s-polarized, multi-resonances are created when the beam reflects back to the dielectric layer after approaching the grating, leading to high absorption (above 90%) over a broad wavelength range of 0.4-1.4 *µ*m. For p-polarized incidence, we can see that absorption decreases rapidly and becomes smaller than 25% for wavelength range of 1.2–1.6 *µ*m. Furthermore, the impedance matching (6) for the absorber under the s-polarized light is observed through the electric field distribution *E*_*s*_ under normal incidence at the wavelength of 600 nm as shown in Fig. 3c, where there is no distortion and change in shape of the wavefront as the incident wave propagates into the silica layer of the absorber from the air, meaning that there is no reflection from the absorber. The time-averaged power flow $\overrightarrow{P}$ (arrows) keeps the same intensity in the air and the top silica layer but decays rapidly below the Cr layer, illustrating the role of the Cr layer whose absorption loss induces the perfect absorption of the incident light.

**Figure 4:**
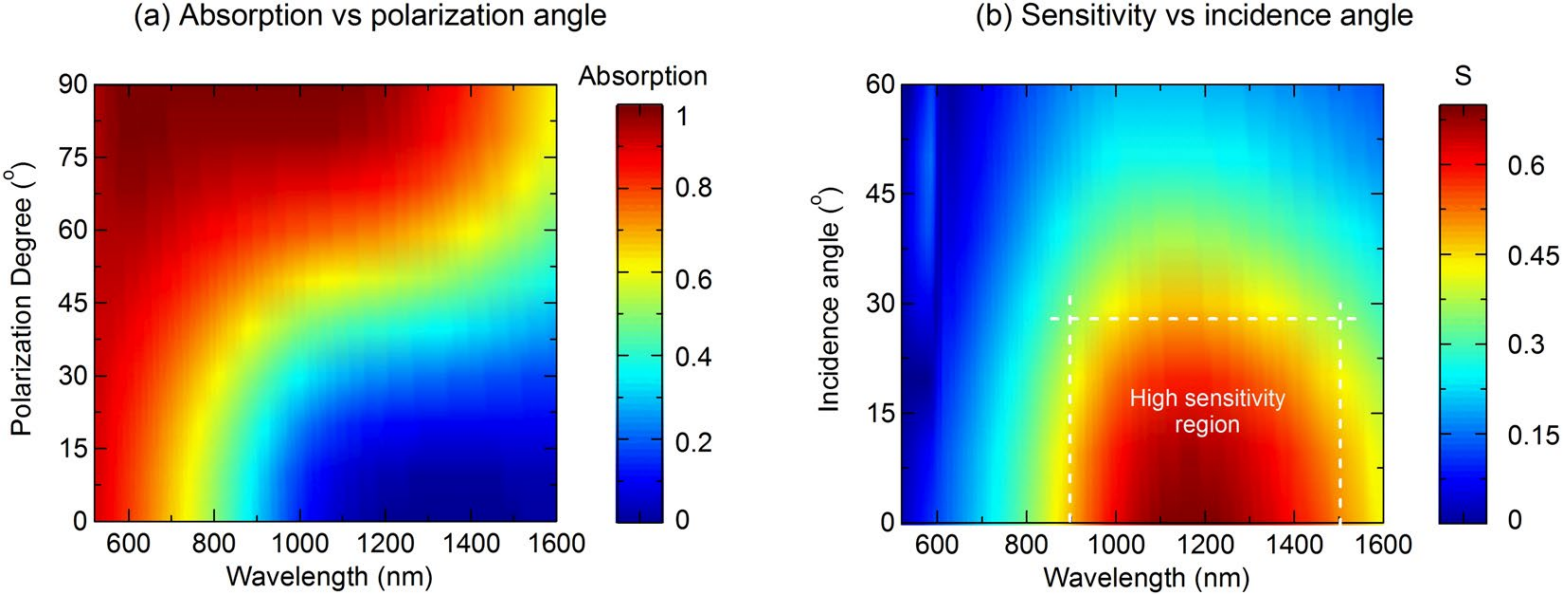
False color plot of absorption spectrum as a function of polarization angle under normal incidence (**a**) and sensitivity as a function of incidence angle (**b**) of the broadband absorber. p-polarization and s-polarization correspond to polarization angles of 90° and 0° respectively.

Figure 4a shows a false-color plot of the absorption as a function of polarization angle, where 0° corresponds to the p-polarization and 90° corresponds to the s-polarization, over broad range of wavelength in visible and NIR regime. We can see that the absorption is high (above 80%) and insensitive to the polarization for wavelength below 700 nm, and becomes sensitive to the polarization for the higher wavelength range, in which the absorption can be tuned from extremely high absorbing state (above 98%) to low absorbing state (below 10%) by rotating the polarization angle from s-polarization (90°) to p-polarization (0°). To identify the sensitivity of the absorber to the polarization, we define a sensitivity parameter S as a difference of the absorption between the s-and p-polarization. Physically, higher S means the absorber is more sensitive to the polarization. An ideal polarization sensitive perfect absorber will have *S* = 1, and a polarization independent perfect absorber has *S* = 0. We perform investigation of the sensitivity as a function of incidence angle and the results are shown in Fig. 4b. In this figure, the hot color corresponds to high sensitivity. This illustrates a high sensitivity region with S above 50% in the wavelength range of 0.9–1.5 *µ*m and incidence angle between 0° and 30°. The sensitivity decreases with larger incident angle and have maximum at normal incidence.

**Figure 5:**
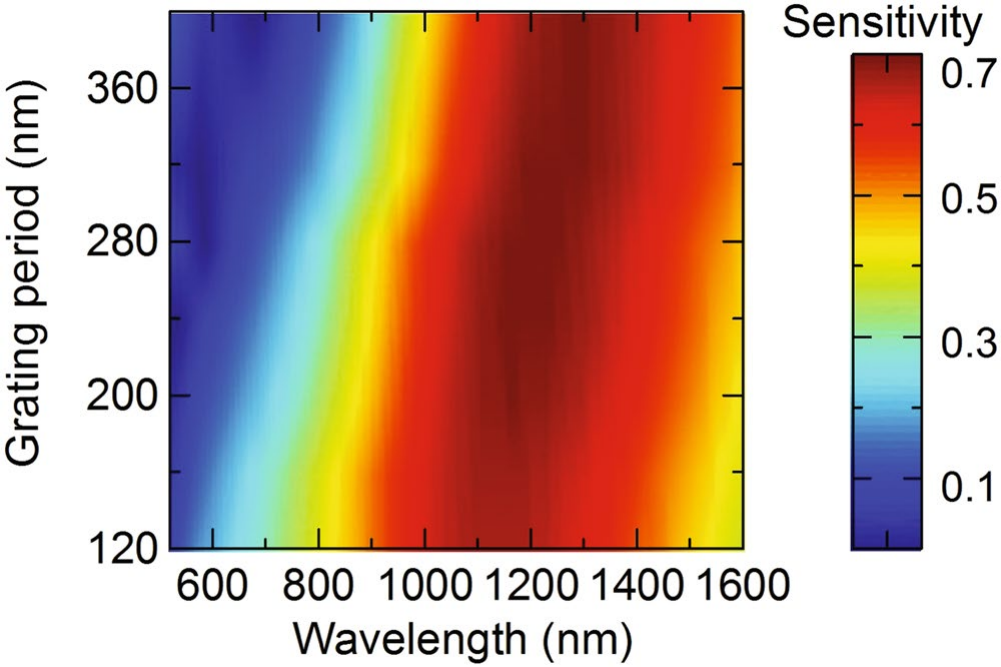
Polarization sensitivity of the broadband absorber as a function of grating period.

To study the performance of the designed absorber to the tolerance of plasmonic grating parameters, we investigate the polarization sensitivity of the absorber as a function of grating period as shown in Fig. 5. Other parameters of the grating such as filling factor and thickness are fixed. For wavelength range of 800–1500 nm, the polarization sensitivity is nearly unchanged when the grating period is varied from 120 nm to 400 nm. This shows that the proposed broadband polarization sensitive perfect absorber does not require a strict value of the grating period, thus illustrating the feasibility for fabrication and realization.

## Narrowband polarization sensitive absorber

Generally, narrowband perfect absorber is based on the extraordinary absorption of plasmonic resonances. A variety of structures has been investigated for selective light absorption, and the metal-insulator-metal (MIM) structure with one side patterned layer has been recently applied for realization of such absorbers. For example, a triple-band perfect absorption in a MIM structure has been demonstrated in ref. 23 by utilizing the coupling between the surface plasmon polariton and the guided mode resonance.

**Figure 6:**
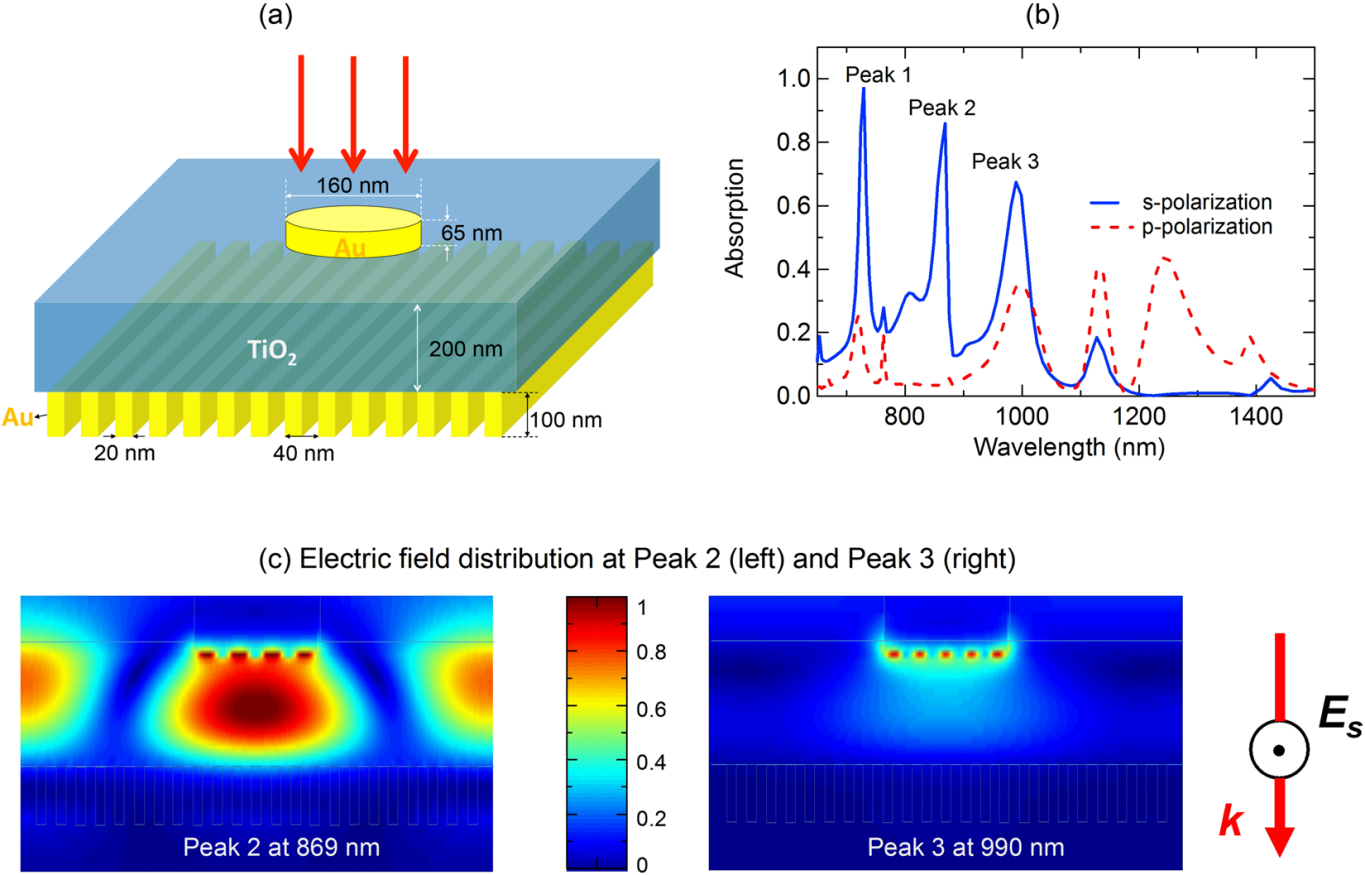
(**a**) Schematic of the narrowband polarization sensitive perfect absorber. Au nano-disk has diameter of 160 nm and thickness of 65 nm. TiO2 layer has thickness of 200 nm, and the Au grating has period of 40 nm, with filling factor of 0.5 and thickness of 100 nm. (**b**) Absorption for s-and p-polarization under normal incidence. (**c**) Electric field distributions at Peak 2 (left) and Peak 3 (right) for s-polarization.

We now demonstrate that a narrowband perfect absorber sensitive to polarization can be realized by using one side patterned metal-insulator-grating (MIG) structure, in which the top layer has patterned plasmonic structure. Figure 6a presents our design for this kind of absorber in the visible and NIR range. A periodic disk structure having radius of 80 nm, thickness of 65 nm and period of 600 nm is patterned on a titanium dioxide (TiO_2_) spacer of thickness of 200 nm. The grating structure has a period of 20 nm and thickness of 100 nm. For s-polarized light with normal incidence, three absorption peaks, denoted as Peak 1 (absorption above 98%), Peak 2 (absorption above 85%) and Peak 3 (absorption above 68%), corresponding to the wavelength of 729 nm, 870 nm and 990 respectively are observed. Peak 1 originates from the loss of Au enhanced by the Fabry-Perrot resonance whose cavity length is equal to the thickness of the spacer layer^23^. The physical mechanism of the absorption Peak 2 and 3 are explored through their electric field distributions as shown respectively in Fig. 6c. The absorption Peak 2 is induced from the coupling between the surface plasmon polaritons and guided mode resonances, and Peak 3 originates from the localized surface plasmon on nano Au discs, in which the position of Peak 3 depends on the size of the nano gold particles. When the p-polarized light is applied, we can see that all of these 3 absorption peaks are diminished as can be seen in Fig. 6b. In particular, the absorption Peak 1, Peak 2 and Peak 3 are changed from 99%, 90% and 70% to below 26%, 5% and 35% respectively when changing the incident light from s-polarization to p-polarization.

**Figure 7:**
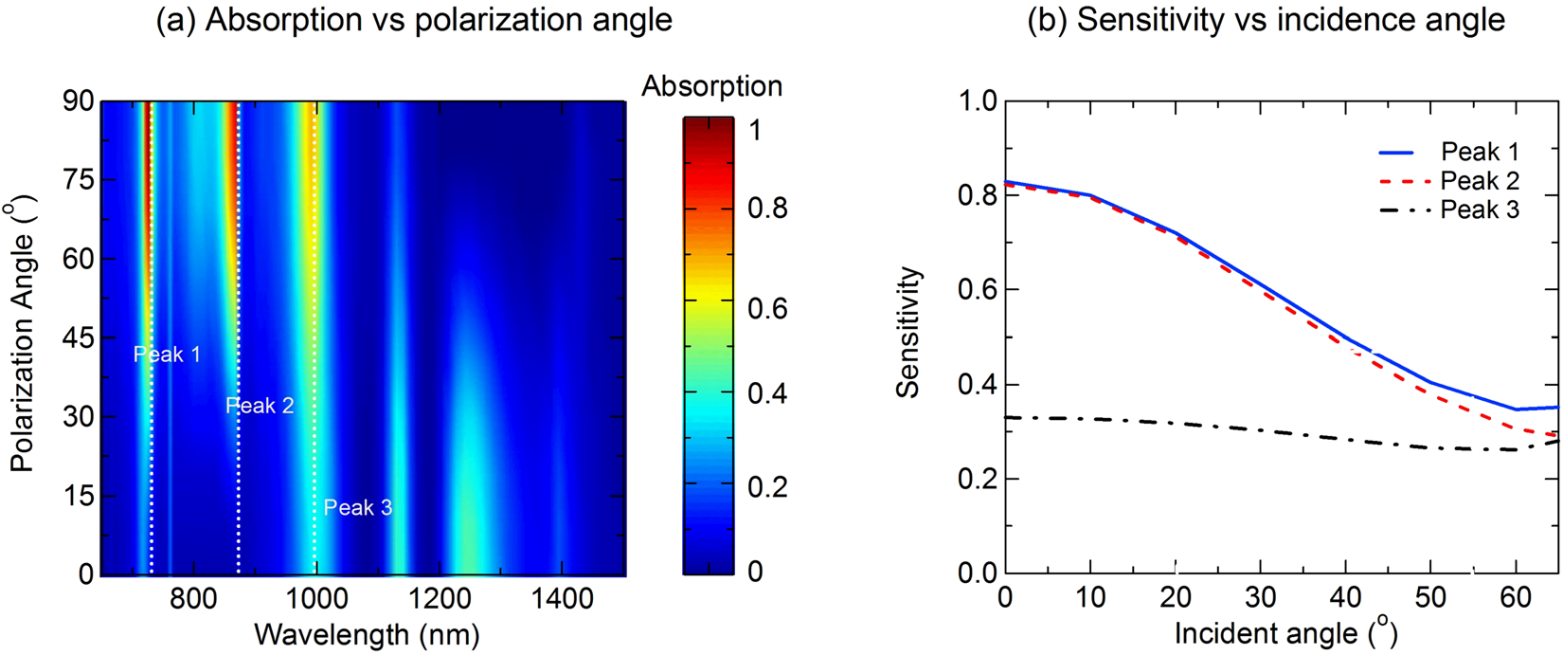
(**a**) Absorption spectrum as a polarization angle under normal incidence and (**b**) sensitivity of triple absorption band as a function of incidence angle of the narrowband absorber.

**Figure 8:**
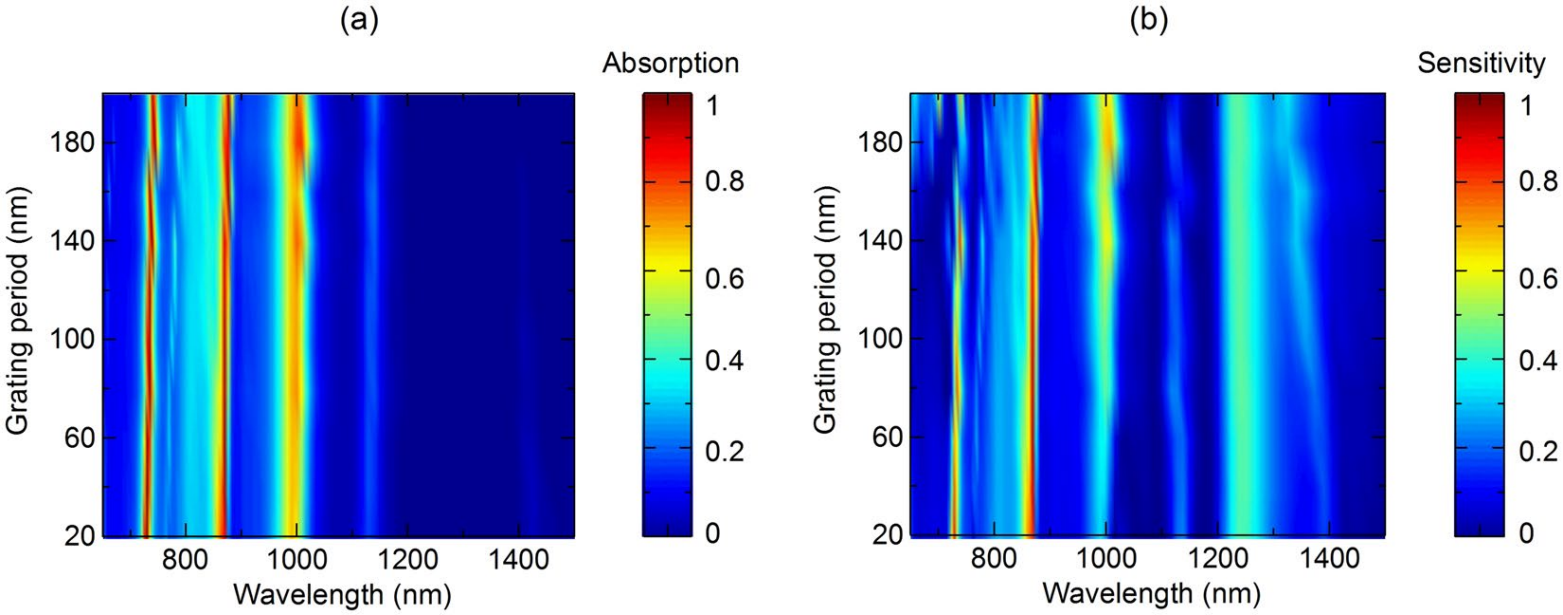
(**a**) Absorption spectrum for s-polarization and (**b**) polarization sensitivity spectrum of the narrowband absorber as a function of the grating period.

Figure 7a demonstrates the ability for active control of the absorption at Peak 1, Peak 2 and Peak 3 by varying the polarization of the incident light, in which we can achieve any expected values of light absorption by setting an appropriate polarization state between 0° (p-polarization) and 90° (s-polarization). The simulation is done under normal incidence. The dependence of polarization sensitivity of the triple-band absorption corresponding to Peak 1, Peak 2 and Peak 3 is shown in Fig. 7b. The results show that the sensitivity of Peak 1 is nearly same as the one of Peak 2, where the sensitivity is highest (0.82) at normal incidence and decreases at larger incidence angle, while the sensitivity of the Peak 3 remains unchanged at low value around 0.33. High sensitivity of absorption Peak 1 and Peak 2 according to the polarization can therefore have potential applications in optical filters, polarization detectors and sensors. We investigate the dependence of the narrowband absorber on the tolerance of the grating period. As shown in Fig. 8, the absorption spectrum for s-polarization (Fig. 8a) and the sensitivity spectrum (Fig. 8b) under normal incident angle are presented for a broad range of grating period of 20–200 nm. We can see that for s-polarization, the first two absorption bands corresponding to Peak 1 (wavelength of 728 nm) and Peak 2 (wavelength of 870 nm) have high absorption that is above 90% for broad range of grating period from 20 nm to 200 nm. However, the third absorption band has lower absorption, and only achieve higher value that is above 80% for grating period larger than 160 nm. Regarding polarization sensitivity, which is defined as the difference of absorption between the s-polarization and p-polarization, false color plot of its spectrum in Fig. 8b illustrates a high independent sensitivity (>80%) of the second absorption band on the grating period. Meanwhile, polarization sensitivity of the first absorption band is higher at smaller grating period, and the one of the third absorption band is higher at larger grating period. These investigations are important to design a polarization sensitive perfect absorber adapting to a specific application whose working wavelength is at absorption band 1, 2 or 3.

## Practical Aspect

**Figure 9:**
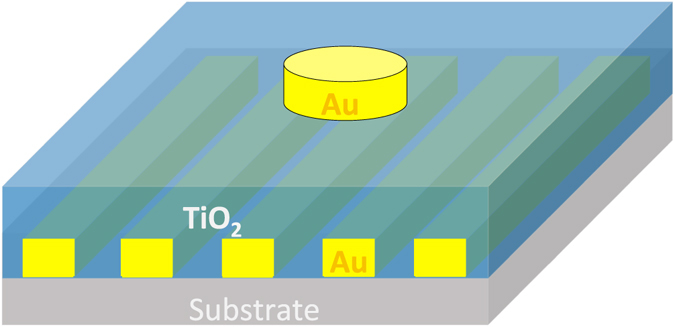
Alternative design of narrowband absorber for realistic fabrication.

We elaborate on the feasibility of fabrication of our plasmonic grating based polarization sensitive perfect absorbers. The broadband perfect absorber depicted in Fig. 3a can be fabricated by depositing respectively Cr and SiO_2_ layers on the SiO_2_ substrate by using the electron beam (e-beam) evaporation. Since the resolution of a commercial e-beam evaporator is less than 1 nm, it would be practical to control precisely the layer thickness. Gold grating will be finally fabricated on the SiO_2_ layer with patterning using e-beam lithography and lift-off process. As explained in Fig. 5, the polarization sensitivity is nearly unchanged when changing the grating period in a broad range from 120 nm to 400 nm. Consequently, it is acceptable for a small difference between the fabricated grating period and the designed, leading to a practical implementation of the designed structure to practical experiments. Regarding the narrowband polarization sensitive perfect absorber, a schematic design for practical fabrication is shown in Fig. 9. Gold gratings will be fabricated first on a silica substrate with patterning using e-beam lithography and lift-off process. Then the TiO_2_ layer is deposited by e-beam evaporation. Au discs are finally placed on the TiO_2_ layer. Since our narrowband absorber bases on the patterned metal-insulator-reflector structure, the absorption properties of the structure are mainly governed by the patterned plasmonic layer and thickness of the insulator providing the condition that the reflector layer has high reflection efficiency. Consequently, the influence of the silica substrate below the grating and the incorporating TiO_2_ in Au grating on the absorption performance of the device in comparison with the design in Fig. 6 is negligible.

## Conclusions

In conclusion, we have demonstrated that a conventional polarization-insensitive multilayer perfect absorber can be switched to a highly polarization-sensitive perfect absorber by replacing the reflector layer, which is normally a metallic thin layer, with a plasmonic grating. We proposed designs of polarization sensitive perfect absorber for both broadband and narrowband of wavelength, and numerically demonstrated the tunability of the light absorption when changing the light polarization. Particularly, light absorption in broadband or triple-narrow band of wavelength obtains highest values (>90%) at s-polarization and decrease rapidly when changing to p-polarization. This effect could be exploited to realize broadband or selective band optical switches controlled by the incident polarization. Furthermore, we showed that the broadband absorber and the second absorption band of the narrowband absorber have good performance over a wide range of grating period, while for the narrowband absorber, the first absorption band prefers shorter grating period and the second absorption band prefers longer grating period. Finally, we discussed the possible implementation of the designs to real fabrication and applications. We foresee that the actual implementation of the plasmonic grating based polarization sensitive perfect absorber may result in a new class of active control of light absorption devices that offer manypromising applications in photovoltaic industry, radar technology, solar cells and thermal imaging.
